# Parental use of structure-based and autonomy support feeding practices with children with avid eating behaviour: an Ecological Momentary Assessment study

**DOI:** 10.1186/s12966-025-01768-x

**Published:** 2025-05-28

**Authors:** Katie L. Edwards, Abigail Pickard, Claire Farrow, Emma Haycraft, Moritz Herle, Clare Llewellyn, Helen Croker, Alice Kininmonth, Jacqueline Blissett

**Affiliations:** 1 School of Psychology and Institute of Health and Neurodevelopment, Aston University, Birmingham, UK; 2 School of Psychology, University of Birmingham, Birmingham, UK; 3 Department of Clinical Psychology, School of Health in Social Science, University of Edinburgh, Edinburgh, UK; 4 School of Sport, Exercise and Health Sciences, Loughborough University, Loughborough, UK; 5 Social, Genetic & Developmental Psychiatry Centre, Institute of Psychiatry, Psychology & Neuroscience, King’s College London, London, UK; 6 Research Department of Behavioural Science and Health, Institute of Epidemiology and Health Care, University College London, London, UK; 7 World Cancer Research Fund International, London, UK; 8 School of Food Science and Nutrition, University of Leeds, Leeds, UK; 9 Leeds Institute for Data Analytics, University of Leeds, Leeds, UK

**Keywords:** Food parenting practices, Preschool children, Avid eating, Health-related goals, Eating occasions, Ecological momentary assessment

## Abstract

**Background:**

Avid eating is an eating profile which confers greater risk for childhood obesity and can be challenging for parents to manage. Using Ecological Momentary Assessment (EMA), we have previously shown that parental mood, feeding goals, and eating context determine parents’ use of coercive and indulgent feeding practices. Parents have also reported using specific noncoercive practices which provide structure (e.g., modelling) or autonomy support (e.g., nutrition education) when feeding children with avid eating behaviour more effectively. However, research is yet to examine the momentary predictors of these adaptive feeding practices.

**Method:**

This EMA study aimed to examine parental mood, goals, and context as momentary predictors of parents’ use of noncoercive feeding practices during daily feeding interactions with preschool children (3–5-years-old) with an avid eating profile. Parents (*N* = 109; females n = 85) completed a 10-day EMA period which assessed momentary mood, feeding goals, feeding practices, and contextual factors.

**Results:**

Parents were more likely to use structure-based feeding practices when feeding goals were health-related, the atmosphere was positive or neutral, or when parents initiated eating occasions. Parents were also more likely to use autonomy support feeding practices when their feeding goals were health-related or when parents initiated eating occasions. Encouraging children to eat or negotiating with children about *how much* or *what* food to eat was significantly associated with a negative atmosphere during eating occasions.

**Conclusions:**

Together, our findings show that parental mood, feeding goals and context are momentary predictors of parents’ use of noncoercive feeding practices to manage children’s avid eating behaviour. Further work is needed to examine whether supporting parents to prioritise health-related goals at mealtimes increases the use of adaptive, noncoercive feeding practices.

**Supplementary Information:**

The online version contains supplementary material available at 10.1186/s12966-025-01768-x.

## Introduction

Approximately one in five (~ 20%) UK preschool children have an avid eating behaviour profile, which is characterised by higher levels of food enjoyment and responsiveness to food cues, overeating in response to negative emotions, faster eating, poorer sensitivity to satiety signals, and lower levels of food fussiness (see Pickard et al., 2024 for more details) [1]. These appetite traits have high genetic and environmental influence; thus, an avid eating behaviour profile confers greater risk for obesity due to complex interactions between genetic susceptibility and exposure to an ‘obesogenic’ environment [2]. Indeed, children’s appetite avidity is prospectively associated with adiposity [3]. Thus, there is a need for tailored health interventions to support parents with managing children’s avid eating behaviour.

Parental feeding practices, which shape the development of children’s eating behaviour, can be described using four overarching domains: coercive feeding practices including (a) coercive control, such as restriction of food, and (b) indulgent, such as allowing children excessive freedom over their food decisions; and noncoercive feeding practices including (c) structure, such as food availability and accessibility, and (d) autonomy support or promotion, such as involving children in food-related decision making [4]. Parental feeding practices appear to be state-dependent, changing over time and context [5]. Since ‘in the moment’ changes cannot be examined in cross-sectional studies, Ecological Momentary Assessment (EMA), a methodology which captures inter- and intra-individual variability in behaviour across time and contexts, presents a useful approach for investigating the momentary predictors of parent-child feeding interactions. Research using EMA has shown that parents of preschool children use a variety of feeding practices depending on the feeding context [5]. Indeed, our EMA research has shown that parents of children with avid eating behaviour are more likely to use coercive or indulgent feeding practices during feeding interactions with a negative atmosphere, in a public setting, and when children are eating a snack (versus meal) [6]. In addition to contextual factors, parental mood and feeding goals were also found to influence feeding practices, whereby parents were more likely to use coercive or indulgent feeding practices when experiencing higher levels of stress and when aiming to avoid conflict [6]. These findings highlight momentary parental and contextual predictors of coercive and indulgent feeding practices; however, these practices are best avoided, because they may exacerbate children’s avid eating behaviour [7]. In contrast, adaptive, noncoercive feeding practices, such as those which provide structure or support children’s autonomy, may be used to promote the development of healthy eating behaviour. For example, using structure-based feeding practices, such as providing a greater balance and variety of food, could be used to harness high levels of food enjoyment and low food fussiness [1]. Indeed, parents report using these noncoercive feeding practices, such as those which provide structure (e.g., modelling) and promote autonomy (e.g., nutrition education), to manage children’s avid eating behaviour [1, 8, 9]. Whilst EMA research has shown that parents are more likely to use structure-based feeding practices at mealtimes and during feeding interactions with a positive mealtime atmosphere [5], there has been no examination of the momentary predictors of the use of these feeding practices by parents of children with avid eating behaviour. Improving our understanding of the momentary factors which influence parental use of noncoercive feeding practices with children who have avid eating behaviour is key to designing tailored interventions to support parents to improve healthy eating and reduce obesity risk in this group of particularly vulnerable children.

Managing children’s avid eating behaviour has been reported as challenging for parents [9]. Our qualitative research findings have illustrated that while parents provide a structured and autonomous food environment for children with avid eating behaviour, they are also more likely to use coercive and indulgent feeding practices, such as using food as a reward, when experiencing time constraints and high stress [9]. This has also been shown in our EMA research, whereby parents are more likely to use coercive and indulgent feeding practices during feeding interactions with children with avid eating behaviour when parents are experiencing high levels of stress and negative mood [6]. Thus, in this paper, we will examine the possibility that parents are more likely to use noncoercive feeding practices which promote structure and autonomy support in situations of positive mood and low stress. Establishing the relationship between fluctuations in parental mood and the use of specific adaptive feeding practices will help to identify whether emotion/mood regulation might be a useful target to support parents with managing children’s avid eating behaviour.

Parental feeding goals also shape the use of feeding practices, and these are likely to vary according to the eating context (e.g., whether a parent or child initiated an eating occasion), and parental stress and affect. Findings from survey data have shown that parents of children with avid eating are more likely to report goals of avoiding mealtime stress and conflict compared to children with ‘happy’ eating behaviour [10]. Moreover, EMA research has shown that parents of children with avid eating behaviour are more likely use coercive or indulgent feeding practices when their feeding goal is to avoid mealtime conflict [6]. Together, these findings suggest that ‘in the moment’ feeding goals predict parental use of coercive feeding practices, thus, here we examine whether feeding goals also predict use of adaptive, noncoercive feeding practices, which may be used to manage children’s avid eating behaviour more effectively.

In summary, parental use of structure-based and autonomy support feeding practices could help to support the development of healthy eating behaviour by children with avid appetites, who are at greater risk of developing obesity. Previous research which has relied on cross-sectional methods does not capture the variability in feeding interactions between parents and children with avid eating behaviour. An improved understanding of momentary influences on noncoercive parental feeding practices will support the development of tailored feeding guidance for parents of children with avid eating behaviour. Therefore, using EMA, this study aims to examine mood and contextual predictors of parental use of structure-based and autonomy support feeding practices during feeding interactions with preschool children (aged 3–5 years) with an avid eating profile. Based on previous research, the following hypotheses were made:

H1: Parents who report lower levels of stress and negative affect, and greater positive affect, at one measurement occasion will be more likely to use (a) structure-based and (b) autonomy support feeding practices at the following eating occasion.

H2: Parents who report health-related feeding goals will be more likely to report (a) structure-based and (b) autonomy support feeding practices.

H3: Parents who report the atmosphere as positive or neutral during an eating occasion will be more likely to use (a) structure-based and (b) autonomy support feeding practices.

H4: Parents who report initiating an eating occasion (i.e., parents and children deciding together, or the parent alone) will be more likely to report using (a) structure-based and (b) autonomy support feeding practices.

## Method

This EMA study was pre-registered on the Open Science Framework (https://osf.io/yt7nc) and the study protocol has been published in Edwards et al. [11]. All items used in this study can be found on the Open Science Framework (https://osf.io/r6789/files/osfstorage). This study forms part of the APPETItE project (Appetite in Preschoolers: Producing Evidence for Tailoring Interventions Effectively) which examines feeding interactions to better understand children’s avid eating behaviour to inform the development of tailored interventions.

### Participants

**Fig. 1:**
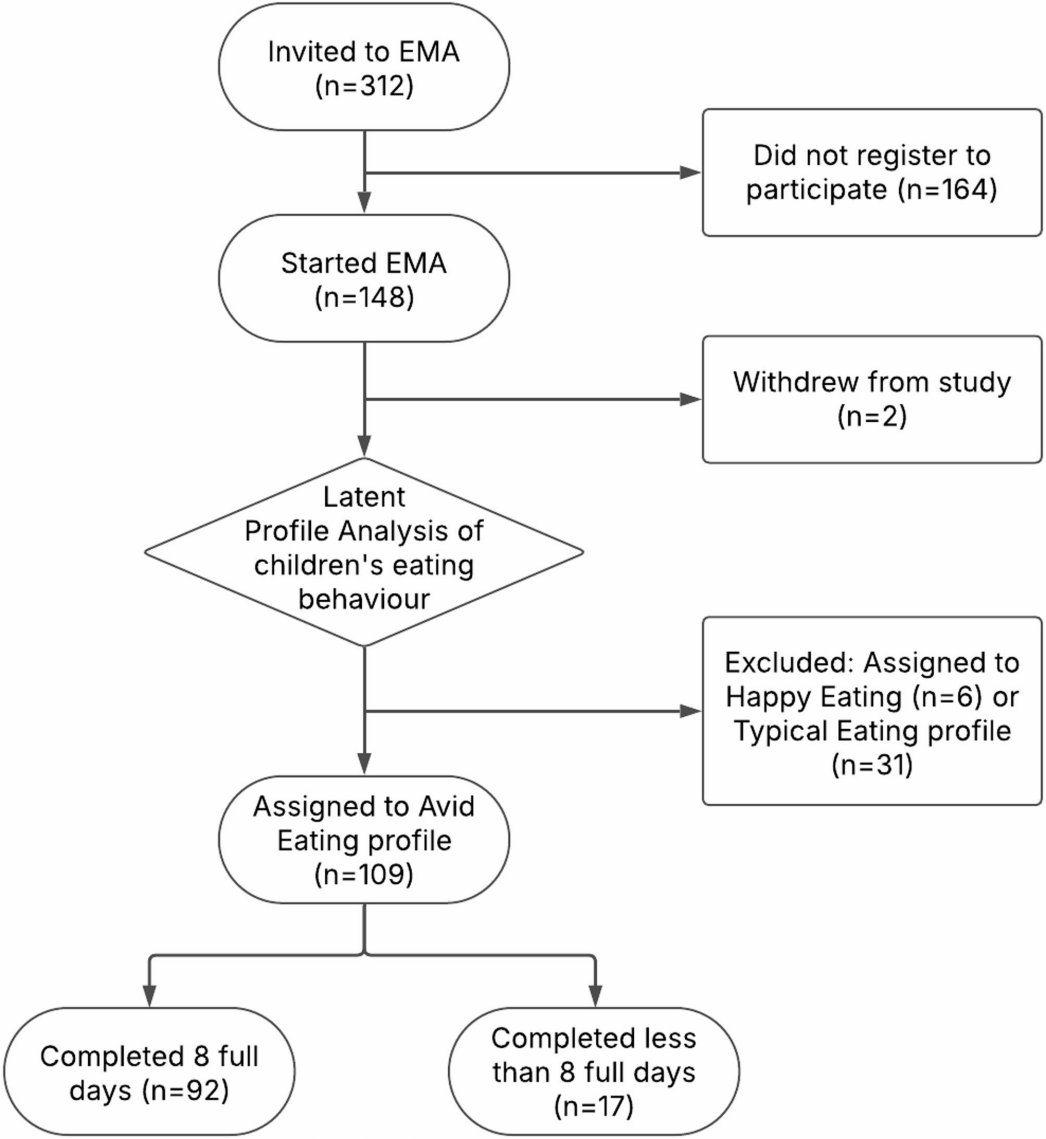
Participant recruitment and retention

Based on previous research [12] we aimed to recruit 200 parents. Whilst a reliable power calculation could not be conducted a priori given the novelty of this EMA study, we had sufficient power to detect meaningful effects. Parents/caregivers of preschool children (3–5 years) who were identified as having an avid eating profile were invited to take part. All participants were from the APPETItE cohort and were invited to take part via email. Data were collected between October 2023 and March 2024. English-speaking primary caregivers (referred to as parents) living in the UK who were responsible for feeding their child for more than half of the time when their child is at home were eligible to participate. Parents whose child was autistic, had severe learning disabilities, or a chronic illness that directly affected their dietary requirements and eating habits were not eligible to participate. In total, 147 parents registered their interest to take part in the EMA study, however, one parent was excluded from data analysis due to their child exceeding the age criteria of 3–5 years (i.e., older than 82 months). Parents’ reports of children’s eating behaviour showed that 109 children had an avid eating behaviour profile. An avid eating behaviour profile was identified using data from the Children’s Eating Behaviour Questionnaire (CEBQ) [13] which identified four distinct eating profiles in preschool children (see Pickard et al., 2023 for more details). Participants with a typical (*n* = 31) or happy (*n* = 6) eating profile were excluded from data analysis. Figure 1 presents full details of participant recruitment and retention. Ethical approval was provided by Aston University Health and Life Sciences Research Ethics Committee (HLS21079). All parents provided informed consent to participate.

### Study design

Parents completed a 10-day period of EMA which assessed momentary parental mood, feeding goals and behaviour, and contextual factors. Seven complete days were needed for this study; however, caregivers are asked to complete a 10-day period to allow flexibility for some incomplete days.

### Procedure

Eligible parents were asked to download an app to their personal smartphone to complete the EMA surveys. Firstly, parents completed a baseline questionnaire which gathered information about parent and child demographics, socioeconomic background, and food security. Parents also provided information about their general mood and wellbeing. Descriptive statistics from the baseline questionnaire have been reported in Pickard et al. [14]. Next, parents completed a 10-day period of EMA which included signal-contingent ‘mood’ surveys, event-contingent ‘food’ surveys, and an end-of-day survey (see Sect. 2.4). All EMA surveys took less than 5 min to complete. Finally, parents were asked to complete an end-of-study questionnaire which assessed the feasibility and usability of completing the EMA study (see Pickard, Edwards [15] for findings). After taking part, parents were sent a debrief form and received a £100 online shopping voucher if they completed at least 8 days of EMA surveys. Parents who did not complete 8 days of EMA (*n* = 17) were reimbursed pro-rata with £10 per complete day. One ‘complete’ day of EMA was defined as the completion of 2 signal-contingent surveys and 1 event-contingent survey. Parents who completed 10 days of EMA were also entered into a prize draw to win an additional £100 online shopping voucher.

### EMA period

#### Signal-contingent surveys

Parents received four semi-random notifications throughout the day to complete a mood survey (signal-contingent) which assessed parental positive and negative affect, stress, and contextual factors. Items measuring positive affect (6 items, e.g., ‘I feel cheerful’) and negative affect (7 items, e.g., ‘I feel annoyed’) were adapted from the Positive and Negative Affect schedule [16] which has been used widely in EMA research [17]. Items measuring parental stress (4 items e.g., ‘I feel tense’) were adapted from the Perceived Stress Scale [18] for use in EMA research [19, 20]. Item responses were on a 5-point Likert scale: 1 = not at all, 2 = a little, 3 = moderately, 4 = quite a bit, 5 = extremely. Mood surveys also included 3 items to examine the context in which surveys were completed; items asked parents where they were, who they were with, and what they were doing (adapted from the PsyMate standard assessment protocol) [21]. The first mood survey of the day (morning survey) also examined parents’ health-related and stress/conflict avoidance feeding goals to determine how these vary throughout the day. Items were adapted from the Family Mealtime Goals Questionnaire (7 items, e.g., ‘To give my child food that is nutritious’) [22]. See Edwards et al. [11] for details of the semi-random notification schedule used for signal-contingent surveys.

#### Event-contingent surveys

Parents completed a food survey (event-contingent) after each time their child ate (‘food consumed’ survey) or asked for food (‘food request’ survey) when their parent was present. These surveys were self-initiated by parents since it was not known when children would eat or ask for food. Food surveys assessed parental feeding practices, goals, and the context of the feeding interaction. Questions were adapted from the Real-Time Parent Feeding Practices measurement tool [23] to examine structure-based, autonomy support, coercive control, and indulgent feeding practices (‘food consumed’ surveys only). Food consumed surveys included 6 items assessing structure-based feeding practices (e.g., ‘choose what foods your child got to eat’) and 8 items assessing autonomy support (e.g., ‘involve your child in deciding what foods they would eat’). To assess why parents did not allow children to have food, food request surveys assessed structure-based feeding practices (4 items, e.g., ‘they asked before a mealtime’) and autonomy support practices (5 items, ‘Teach your child about why you wanted them to eat less of certain foods’). For brevity, coercive and indulgent feeding practices will not be discussed further in this paper since they have been reported elsewhere [6]. Food surveys also examined mealtime feeding goals adapted from Snuggs et al. [22], including health-related goals (e.g., ‘To give my child food that was nutritious’) and stress/conflict avoidance goals (e.g., ‘To avoid arguments about food at mealtimes’). Responses were ‘yes’ or ‘no’, with a ‘not applicable’ option for items about parental feeding practices. Additionally, parents were asked what their main feeding goal was out of the items presented and had a free-text option to report their own feeding goal. Food surveys also examined the context of the feeding interaction including who was present, the location, what the atmosphere was like, and the type of food asked for or eaten (see Table 1 for response options). Items and response options were adapted from Trofholz et al. [19]. Food consumed surveys also asked who initiated the eating occasion.

**Table 1:** EMA response options and coding scheme used for analysis

	Response Options	Dummy Code
Health-related feeding goals *		
To give my child food that was nutritious	1 = Yes	Yes
	0 = No	**No**
I wanted my child to be free to eat unhealthy food	1 = Yes	**Yes**
	0 = No	No
To ensure my child has a balanced diet overall	1 = Yes	Yes
	0 = No	**No**
**Meal Context**		
Meal child was eating	1 = Breakfast	Meal
	2 = Lunch	Meal
	3 = Evening meal	Meal
	4 = Snack	**Snack**
	5 = Meal at large family gathering	Meal
Initiator of eating occasion	1 = Parent	Parent
	2 = Child	**Other**
	3 = Parent and child	Parent
	4 = Partner	**Other**
	5 = Other child	**Other**
	6 = Other family member	**Other**
	7 = Non-family member	**Other**
Atmosphere of eating occasion	1 = Chaotic	**Negative**
	2 = Rushed	**Negative**
	3 = Tense	**Negative**
	4 = Relaxed	Positive
	5 = Enjoyable	Positive
	6 = Neutral	Positive

**Note**. For each variable, parents could select one response item only. Items in bold indicate reference variables. Reference variables were not theoretically predicted to be associated with parental use of structure-based or autonomy support feeding practices. *A composite health-related goal score was calculated as the sum of the 3 health-related goal items. Higher scores indicate greater endorsement of health-related goals

### Data analysis

Data were cleaned and coded in R version 4.4.1. by AP and KLE and were independently checked by AK. Data were analysed by KLE. SPSS Version 29 was used to conduct descriptive statistics to characterise the sample and eating occasions. Multi-level models were conducted in R version 4.4.1 with the lme4 package [24] to examine the study’s hypotheses. To examine temporal ordering, data from eating surveys were paired with data from mood surveys that were completed up to 4-hours earlier on the same day for each participant. Outcome variables were structure-based and autonomy support feeding practices (see Table 2). These variables were treated as dichotomous and were run in separate multi-level models. Parent stress, negative affect, and positive affect were centred for each participant. Categorical items were re-coded and included in the model as binary variables to maintain sufficient sample sizes (see Table 1). As pre-registered, models were adjusted for the following covariates: parent age, child age, the probability of children’s avid eating behaviour, and whether the eating occasion occurred on a weekday or weekend. Multi-level models included a random intercept that was allowed to vary within individuals and a random slope that was allowed to vary within individuals. *P*-values were used to determine whether models were significantly different from zero (*p* <.05). This analytic approach has been used in our prior EMA research [6] and is like the approach used in other EMA research which has used similar measures e.g., Loth et al. [5].

**Table 2:** Structure-based and autonomy support feeding practices

***Structure***	
SPFP1	Sit and eat with your child (modelling)
SPFP2	Choose where your child ate meal or snack (meal & snack routines)
SPFP3	Choose what foods your child got to eat (food availability)
SPFP4	Closely monitor the type of food eaten by your child (monitoring-type)
SPFP5	Closely monitor the amount of food eaten by your child (monitoring-amount)
SPFP6	Allow your child to choose what to eat, from several options you picked out (guided choices)
***Autonomy Support***	
APFP7	Involve your child in deciding what foods they would eat (child involvement-what)
APFP8	Allow your child to take more food if they asked for it (child involvement-more)
APFP9	Teach your child about why you wanted them to eat more of certain foods (nutrition education-why more)
APFP10	Teach your child about why you wanted them to eat less of certain foods (nutrition education-why less)
APFP11	Encourage your child to try at least a small amount of all foods offered (encouragement)
APFP12	Negotiate with your child about how much food to eat (negotiation-amount)
APFP13	Negotiate with your child about what food they eat (negotiation-what)
APFP14	Tell your child you wanted them to eat less of certain foods (reasoning)

**Note**. SPFP = Structure-based parental feeding practice; APFP = Autonomy support parental feeding practice

## Results

### Sample characteristics

Parents (*N* = 109) were mostly females (*n* = 85, 78%) with a mean age of 34.6 years (SD = 5.5, range = 24.8–55.3). Children with an avid eating profile (females *n* = 59, 54%) had a mean age of 53.1 months (SD = 10.3, range = 36.8–71.43). See Table 3 for details of parent demographics.

**Table 3:** Sample characteristics (*N* = 109)

	*n*	%
**Parent Ethnicity**		
Asian	8	7.3
Black	7	6.4
White	91	83.5
Mixed	1	1.0
Other	2	1.8
**Education**		
Degree	64	58.7
No-degree	45	41.3
**Working Status**		
Unemployed	21	19.3
Working part-time (between 8–29 h per week)	35	32.1
Working full-time (30 h or more per week)	53	48.6
**Living on Household Income**		
Living Comfortably	47	43.1
Managing	46	42.2
Finding it Difficult	9	8.3
Finding it Very Difficult	6	5.5
**Household Food Security**		
High or Marginal Food Security	72	66.1
Low Food Security	16	14.7
Very Low Food Security	21	19.3

**Note**. Household food security was measured using the Household Food Security Scale (Blumberg et al., 1999)

### Eating occasions

**Fig. 2:**
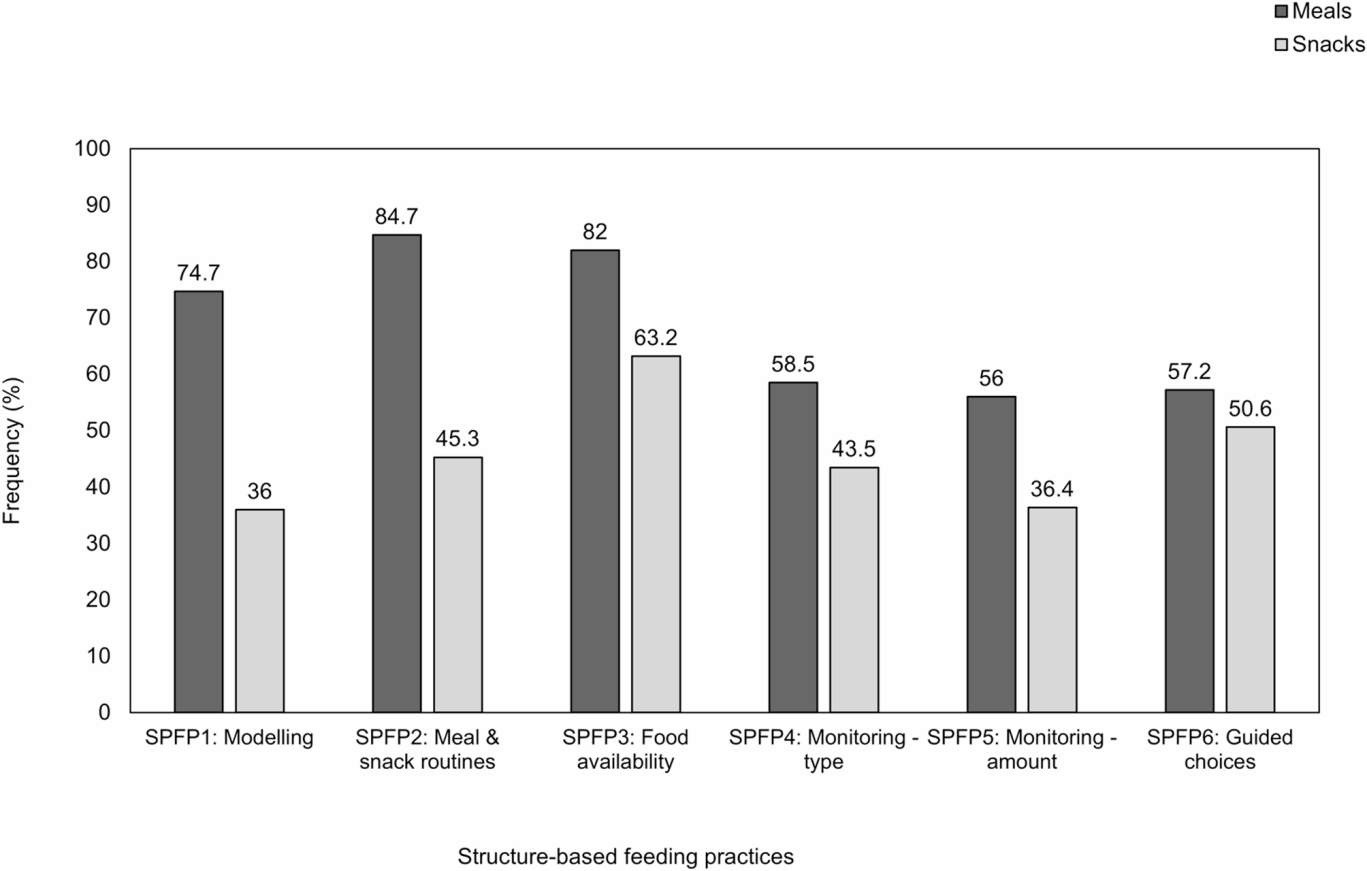
Frequency (%) of parental use of momentary structure-based feeding practices used, split by meal and snack times. Percentages indicate the frequency of parents reporting ‘yes’ to using the specific feeding practice for snacking occasions (n = 506) and mealtimes (n = 1271)

**Fig. 3:**
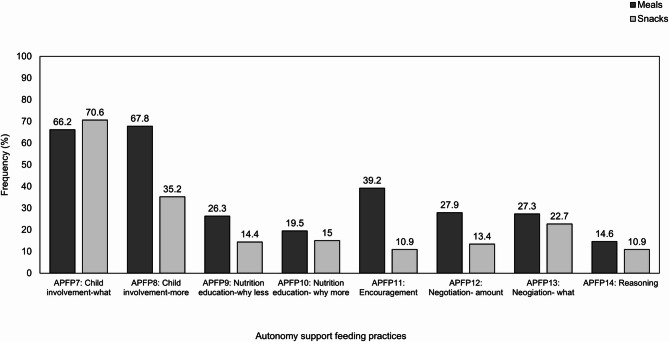
Frequency (%) of parental use of momentary autonomy support feeding practices used, split by meal and snack times. Percentages indicate the frequency of parents reporting ‘yes’ to using the specific feeding practice for snacking occasions (n = 506) and mealtimes (n = 1271)

In-the-moment child eating occasions (*N* = 1777) were reported by parents. Most eating occasions were meals (*n* = 1271, 71.5%) and these were often initiated by parents (*n* = 1011, 56.9%). Parents reported that eating occasions had a typically positive (*n* = 1305, 73.4%), and sometimes neutral (*n* = 230, 12.9%), or negative (*n* = 242, 13.6%) atmosphere. For structure-based feeding practices, choosing what foods children got to eat (SPFP3) was the most reported feeding practice (*n* = 1362, 76.6%). For autonomy support feeding practices, allowing children to take more food if they asked for it (APFP8) was the most reported feeding practice (*n* = 1526, 85.9%). The use of specific parental feeding practices differed significantly between the type of eating occasion, whereby parents were more likely to use structure-based and autonomy support feeding practices for mealtimes, compared to snack times (all *p*’s < 0.05). However, negotiating with children about what food they eat (APFP13) did not differ significantly between meal and snack times (*p* =.057). See Figs. 2 and 3, and Table A in Additional File 1 for the frequency of parental feeding practices.

### Momentary effects

Multi-level models demonstrated effects of stress, affect, feeding goals, and context effects (mealtime atmosphere, who initiated the eating occasion, meals vs. snacks, weekend vs. weekday) and individual effects (child probability of avid eating, parent and child age) on parent use of structure-based (SPFP) and autonomy support feeding practices (APFP). Table 4 illustrates the overall model coefficients. Additional Files 2 (tables B-G) and 3 (Tables H-O) provide full model details.

#### Hypothesis 1

*Parents who report lower levels of stress and negative affect*,* and greater positive affect*,* at one measurement occasion will be more likely to use structure-based* and *autonomy support feeding practices at the following eating occasion.*

In support of our hypothesis, parents were significantly more likely to choose where children ate (SPFP2) when they reported greater positive affect or lower negative affect in the 4 h previously. Parents were also significantly more likely to monitor the *type* of food children ate (SPFP4) when they had previously reported greater positive affect. Parental use of encouragement to eat (APFP11) was significantly associated with lower negative affect in the previous 4 h. However, in contrast to our hypothesis, parents who reported greater stress than usual in the 4 h previously were significantly more likely to choose where children ate (SPFP2) or monitor the *type* of food children ate (SPFP4). Moreover, parental use of encouragement to eat (APFP11) was significantly associated with greater stress, or lower positive affect in the previous 4 h. There was no significant relationship between parental stress, positive affect, or negative affect and SPFP1 (modelling), SPFP3 (food availability), SPFP5 (monitoring - amount) or SPFP6 (guided choices), and all other autonomy-support feeding practices.

#### Hypothesis 2

*Parents who report health-related feeding goals will be more likely to report structure-based and autonomy support feeding practices.*

In support of our hypothesis, when feeding goals were health-related, parents were found to be significantly more likely to choose *where* (SPFP2) or *what* children ate (SPFP3) or monitor the *type* of food children ate (SPFP4). Health-related feeding goals were also found to be significantly positively associated with parents allowing children to take more food if they asked (APFP8) or parental encouragement of children to eat (APFP11). However, parents were significantly less likely to sit and eat with children (SPFP1) when feeding goals were health related. There was no significant relationship between health-related feeding goals and SPFP5 (monitoring – amount) or SPFP6 (guided choices), and all other autonomy support feeding practices.

#### Hypothesis 3

*Parents who report the atmosphere as positive or neutral during an eating occasion will be more likely to use structure-based and autonomy support feeding practices.*

As predicted, a positive or neutral atmosphere was found to be significantly associated with parents being more likely to sit and eat with children (SPFP1), choose where children ate (SPFP2), or involve children in what food to eat (APFP7). However, in contrast to our hypothesis, a *negative* mealtime atmosphere during eating occasions was significantly associated with monitoring the *type* of food children ate (SPFP4), parental use of encouragement to eat (APFP11) or negotiating with children about *how much* (APFP12) and *what* food to eat (APFP13). There was no significant relationship between mealtime atmosphere and the following feeding practices: SPFP3 (food availability), SPFP4 (monitoring – type), SPFP5 (monitoring – amount), APFP8 (child involvement – more), APFP9 (nutrition education – why more), APFP10 (nutrition education – why less) or APFP14 (reasoning).

#### Hypothesis 4

*Parents who report initiating an eating occasion (i.e. parents and children deciding together*,* or the parent alone) will be more likely to report using structure-based and autonomy support feeding practices.*

When parents initiated eating occasions, they were significantly more likely to sit and eat with children (SPFP1), choose *where* (SPFP2) or *what* foods children ate (SPFP3), monitor the *type* or *amount* of food children ate (SPFP4 & SPFP5), or encourage children to eat (APFP11). Eating occasions initiated by someone other than the parent was significantly associated with involving children in what foods they ate (APFP7). However, SPFP6 (guided choices) and all other autonomy support feeding practices were not significantly associated with who initiated the eating occasion.

#### Exploratory analysis of the type of eating occasion

We also explored the effect of whether the eating occasion was a meal or snack. When children were eating a meal (rather than a snack), parents were significantly more likely to sit and eat with children (SPFP1), choose where children ate (SPFP2), or monitor the *amount* of food children ate (SPFP5) when children were eating a meal. Additionally, when children were eating a meal rather than a snack, parents were significantly more likely to allow children to have more food (APFP8), encourage children to eat (APFP11), and negotiate with children about *how much* food to eat (SPFP12). In contrast, when children were eating a snack, parents were significantly more likely to monitor the *type* of food children ate (SPFP4).

#### Covariates

Model covariates were significantly associated with structure-based feeding practices. On a weekend day, parents were significantly more likely to sit and eat with children (SPFP1), whereas on a weekday parents were significantly more likely to choose *where* children ate (SPFP2) or monitor the *type* of food children ate (SPFP4). Parents of children with a lower probability of avid eating behaviour were more likely to sit and eat with children (SPFP1), choose where children ate (SPFP2), or monitor the *type* of food children ate (SPFP4). Parents of older children were more likely to sit and eat with children (SPFP1) or guide children’s food choice (SPFP6). Parents of younger children were more likely to choose where children ate a meal or snack (SPFP2) or monitor the *type* of food children ate (SPFP4). Parents who were older were more likely to sit and eat with children (SPFP1), choose where children ate a meal or snack (SPFP2), or monitor the *type* of food children ate (SPFP4).

Model covariates were also significantly associated with autonomy support feeding practices. On a weekend day, parents were significantly more likely to involve children in what foods they ate (APFP7). On a weekday, parents were significantly more likely to encourage children to eat (APFP11). Encouragement to eat (APFP11) was also significantly predicted by greater child age, lower probability of children’s avid eating, and younger parent age.

**Table 4:** Model coefficients for structure-based and autonomy support feeding practices

	Coefficient	Std. Error	t	*p*
**Structure-based**				
SPFP1: Sit and eat with your child	-1.321	0.002	-869.25	**< 0.001**
SPFP2: Choose where your child ate meal or snack	-0.730	0.001	-621.707	**< 0.001**
SPFP3: Choose what foods your child got to eat	1.480	1.208	1.225	0.220
SPFP4: Closely monitor the type of food eaten by your child	-3.496	0.001	-3842.582	**< 0.001**
SPFP5: Closely monitor the amount of food eaten by your child	-1.465	2.218	-0.660	0.509
SPFP6: Allow your child to choose what to eat, from several options you picked	-0.015	0.965	-0.015	0.988
**Autonomy support**				
APFP7: Involve your child in deciding what foods they would eat	1.927	1.404	1.373	0.170
APFP8: Allow your child to take more food if they asked for it	-3.764	2.656	-1.418	0.156
APFP9: Teach your child about why you wanted them to eat more of certain foods	-5.432	2.842	-1.911	0.056
APFP10: Teach your child about why you wanted them to eat less of certain foods	-6.341	3.105	-2.042	**0.041**
APFP11: Encourage your child to try at least a small amount of all foods offered	-1.821	0.002	-1140.918	**< 0.001**
APFP12: Negotiate with your child about how much food to eat	-2.411	1.618	-1.490	0.136
APFP13: Negotiate with your child about what food they eat	-1.699	1.936	-0.878	0.380
APFP14: Tell your child you wanted them to eat less of certain foods	-6.838	3.905	-1.751	0.080

**Note**. SPFP = Structure-based parental feeding practice; APFP = Autonomy support parental feeding practice

## Discussion

This novel Ecological Momentary Assessment (EMA) study examined parental mood, feeding goals and contextual predictors of parents’ use of structure-based and autonomy support feeding practices to manage preschool children’s (aged 3–5 years) avid eating behaviour. Findings showed that parental mood, goals and context predicted parents’ use of a variety of structure-based and autonomy support feeding practices.

There were mixed findings for momentary mood as a predictor of parental feeding practices. For example, whilst the use of monitoring and eating routines was associated with greater parental stress in the preceding 4 h, it was also associated with greater positive affect in that time frame. These findings initially appear contradictory. Previous research has typically found that with increases in parental stress, there is a reduction in monitoring [25]. However, the analysis of ‘higher stress’ in this sample is only relative to the typical experience of each individual within the sample. In other words, the rating of higher momentary stress is actually measuring ‘higher than normal for that individual’. Thus, the description of *higher* stress should not be interpreted as *highly* stressed. The finding may be better interpreted as that when parents have lower levels of stress than usual, they are less likely to use monitoring or eating routines. It may also be the case that parents reporting higher stress who are also able to maintain high levels of positive affect are more resilient, which protects them from reducing their use of monitoring. This highlights the importance of using momentary measures to examine mood as a predictor of parental feeding practices. Overall, our findings suggest that parental mood influences the subsequent use of specific noncoercive feeding practices to manage children’s avid eating behaviour.

Supporting our hypothesis (H2), parental report of health-related feeding goals were positively associated with the use of structure-based feeding practices, such as monitoring, having eating routines, and controlling food availability. Health-related feeding goals were also positively associated with autonomy support practices including parental encouragement to eat and involving children in decisions about food. Whilst having health-related feeding goals appeared to have overwhelmingly positive effects on the subsequent use of noncoercive practices, unexpectedly, parents were found to be *less* likely to sit and eat with their children when their feeding goals were health focussed. One explanation could be that when parents do sit and eat with their children, they prioritise different goals, such as social cohesion through shared family mealtimes, rather than health [22]. Though such goals were not measured in the current study, future research which examines fluctuation in a wider range of feeding goals is needed to better understand the complex motivations underlying parental feeding behaviour. Overall, these findings demonstrate the greater use of noncoercive feeding practices by parents of children with avid eating behaviour when their feeding goals are health focussed (e.g., ensuring that children have a balanced diet). Developing health interventions which align with parents’ feeding goals is critical for encouraging and maintaining behaviour change [22], and further work to establish how best to promote healthy feeding goals is needed.

Findings showed that the atmosphere during feeding interactions predicted parental use of several feeding practices. However, the direction of findings was mixed. Consistent with our hypothesis (H3) and previous EMA research [5], parents were more likely to use structure-based feeding practices, such as having meal and snack routines and modelling eating behaviour, when feeding interactions had a positive or neutral atmosphere. Findings also showed that a negative atmosphere was associated with parental monitoring, encouragement of, or negotiation about children’s eating. Whilst contrary to our hypothesis, this could be explained by the narrow line between practices such as encouragement (e.g., encourage your child to try at least a small amount of all foods offered) and negotiation to eat healthy food (e.g., negotiate with your child about *what* or *how much* to eat), with coercive practices such as pressure to eat (e.g., encourage your child to eat more food than they wanted to). Thus, parent report of their encouragement of, or negotiation with children to eat could actually be the coercive practice of pressure to eat, particularly if delivered repeatedly or with a direct tone [4], which we have previously shown to be associated with negative mealtime atmosphere [6]. Thus, items which examine these feeding practices may need revising to give more nuance and improve parents’ interpretation of what is being asked. This is particularly challenging in EMA methodology, where surveys need to be very quick to complete: items need to be quick to read and take little time to respond to. Capturing subtleties such as the difference between encouragement or negotiation and pressure in this context is challenging. Nonetheless, taken together, these findings highlight the influence of contextual factors (e.g., atmosphere) during daily feeding interactions on parental use of structure-based and autonomy support feeding practices to manage children’s avid eating behaviour.

Consistent with our hypothesis (H4), when parents initiated the eating occasion, they were more likely to use structure-based feeding practices, such as modelling, monitoring, controlling food availability, or having eating routines. These findings are consistent with qualitative research which showed that parents used structure-based feeding practices, such as eating routines, to manage children’s avid eating behaviour [9]. In contrast, we found limited evidence for the effect of parent initiation on the use of autonomy support feeding practices, with only encouragement to eat being predicted by parent’s initiation of eating occasions. This is consistent with the fact that most of the reported eating occasions were meals, which were in the majority initiated by parents, an eating occasion where parents are likely to want the child to eat what is offered. One explanation for the lack of other autonomy support practices being related to parent initiation could be that in general, parent report of using autonomy support feeding practices in this age group was relatively low. This low level of use of autonomy support practices could be due to parents being less likely to use these practices when feeding children with an avid eating profile, or to parents perceiving autonomy of eating as less appropriate for preschool aged children, particularly in the context of mealtimes. Nonetheless, the use of beneficial structure-based feeding practices was more likely when parents are initiating the feeding interaction.

This study also demonstrates important findings about other contextual factors which relate to parents’ use of feeding practices. The use of structure-based and autonomy support feeding practices to manage children’s avid eating behaviour was found to depend on the type of eating occasion (i.e., meal versus snack time). For example, parents were more likely to have eating routines or use modelling, encouragement, or negotiation during mealtimes. Parental monitoring of the *amount* or *type* of food children ate also predicted meal and snack times, respectively. These findings align with previous research showing that parental feeding practices differ by the type of eating occasion [5, 9]. Similarly to previous EMA research [5], parents were also found to use different feeding practices on weekdays versus weekend days. For example, the use of modelling and child involvement during eating occasions was greater on weekend days, whereas the use of eating routines, monitoring, or encouragement was more likely on a weekday. These findings could reflect parents spending more time with children during weekend days, compared to weekdays where children may be in childcare and/or parent time is limited. Overall, parents appear to use different feeding practices across the week, highlighting the importance of examining feeding behaviour across time. These findings also indicate potentially good times or contexts to target within interventions. For example, aspects of interventions which are intended to promote child involvement could suggest parents try suggested activities at weekends, whereas support for reducing excess encouragement to eat could be targeted to weekdays, tackling the additional stresses and pressures associated with those days.

Momentary and contextual factors were not found to predict parents’ use of several feeding practices including guiding children’s food choices, teaching children about nutrition, and reasoning. One explanation could be the lower prevalence of parents using these feeding practices with the children in our sample, who show high levels of food approach behaviours. For example, out of all the reported eating occasions, parents reported teaching children about nutrition and using reasoning for less than 25% of occasions. This suggests that these feeding practices may be less commonly used for managing young children’s avid eating behaviour. Indeed, parents may not have felt the need to use practices such as educating children about nutrition since children with avid eating behaviour do not tend to be selective, and are generally more willing to eat, including nutritious food such as fruit and vegetables [9].

### Implications

The current findings provide important implications for the development of tailored feeding interventions to help parents with managing preschool children’s avid eating behaviour. Supporting and extending previous research [1, 9], our findings show that parents are more likely to use adaptive and noncoercive feeding practices when they have health focussed feeding goals, and during feeding interactions that they initiate themselves. Encouraging parents to use noncoercive feeding practices may be helpful for managing children’s appetite avidity, such as poor satiety responsiveness and emotional overeating. Indeed, structure-based feeding practices are associated with better self-regulation in eating [26] and lower emotional overeating [27]. Based on our findings, the use of structure-based feeding practices during eating occasions with a positive atmosphere may be beneficial for promoting the high levels of food enjoyment observed in an avid eating profile. Indeed, it is important for children to experience positive feeding interactions, such as through positive modelling, for the development of healthy eating behaviour [28, 29]. Overall, our findings suggest that encouraging parental use of noncoercive feeding practices could be a key target in health interventions to support development of children’s healthy eating.

### Strengths and limitations

The use of EMA provides a large amount of novel data about daily feeding interactions between parents and children with avid eating behaviour. EMA allows the examination of variability in feeding interactions across time and context, extending the findings from previous cross-sectional research which has used static questionnaire measures. However, EMA may not capture subtle behavioural nuances because of the nature of the methodology, which relies on brief responses to individual questions. Furthermore, while measures were taken to improve the accessibility of the study, for example, the option to request a smartphone, in-depth study instructions (written and video), and technical support from the research team [11], it is still possible that the intensity of the study and challenges with accessibility were barriers for participation. Though some parents (43%) reported experiencing technical issues when completing the study, overall, EMA presents a feasible and acceptable approach for collecting extensive data about daily feeding interactions with preschool children (see Pickard et al. [15] for more details of the study feasibility).

## Conclusion

This EMA study is the first to demonstrate that momentary fluctuations in parental mood, feeding goals, and the context of eating occasions predicts parents’ use of structure-based and autonomy support feeding practices to manage children’s avid eating behaviour. These findings provide an understanding of the momentary and contextual factors which predict parents’ use of noncoercive feeding practices during daily feeding interactions. This is critical for the development of tailored interventions that provide feeding support for parents to manage children’s avid eating behaviour. Promoting parents’ use of noncoercive practices when feeding children with avid eating behaviour is important for the development of healthy eating behaviour and to minimise the associated obesity risk.

## Electronic supplementary material

Below is the link to the electronic supplementary material.

Supplementary Material 1

## Data Availability

Data are available on the Open Science Framework: https://osf.io/r6789/.
