# Supplementary material for: Parental use of structure-based and autonomy support feeding practices with children with avid eating behaviour: an Ecological Momentary Assessment study
Source: Int J Behav Nutr Phys Act. 2025 May 28;22:66. doi: 10.1186/s12966-025-01768-x (PMC12121011; doi:10.1186/s12966-025-01768-x)
Supplement: Supplementary file 1 — Supplementary Material 1 [file 12966_2025_1768_MOESM1_ESM.docx]

**Additional file 1**

**Table A.** Frequency of parental use of momentary structure-based and autonomy support feeding practices used, split by meal and snack times (Chi-square)

|  | **Total**  (n=1777) | | **Meals**  (n=1271) | | **Snacks**  (n=506) | |  |  |
| --- | --- | --- | --- | --- | --- | --- | --- | --- |
|  | *n* | % | *n* | % | *n* | % | *X^2^* | *p* |
| **Structure-based** | | | | |  |  |  |  |
| SPFP1: Modelling | 1131 | 63.8 | 949 | 74.7 | 182 | 36.0 | 233.56 | **<.001** |
| SPFP2: Meal & snack routines | 1306 | 73.5 | 1077 | 84.7 | 229 | 45.3 | 312.28 | **<.001** |
| SPFP3: Food availability | 1362 | 76.6 | 1042 | 82.0 | 320 | 63.2 | 70.40 | **<.001** |
| SPFP4: Monitoring-type | 964 | 54.2 | 1247 | 58.5 | 220 | 43.5 | 34.96 | **<.001** |
| SPFP5: Monitoring- amount | 896 | 50.4 | 1255 | 56.0 | 184 | 36.4 | 56.35 | **<.001** |
| SPFP6: Guided choices | 983 | 55.3 | 1235 | 57.2 | 256 | 50.6 | 3.15 | **.043** |
| **Autonomy support** | | | | |  |  |  |  |
| APFP7: Child involvement- what | 1198 | 67.4 | 1259 | 66.2 | 489 | 70.6 | 6.29 | **.007** |
| APFP8: Child involvement- more | 1526 | 85.9 | 862 | 67.8 | 178 | 35.2 | 191.34 | **<.001** |
| APFP9: Nutrition education - why more | 407 | 22.9 | 334 | 26.3 | 73 | 14.4 | 28.77 | **<.001** |
| APFP10: Nutrition education - why less | 324 | 18.2 | 248 | 19.5 | 76 | 15.0 | 5.41 | **.011** |
| APFP11: Encouragement | 553 | 31.1 | 498 | 39.2 | 55 | 10.9 | 106.24 | **<.001** |
| APFP12: Negotiation-amount | 423 | 23.8 | 355 | 27.9 | 68 | 13.4 | 35.35 | **<.001** |
| APFP13: Negotiation-what | 462 | 26.0 | 347 | 27.3 | 115 | 22.7 | 2.68 | .057 |
| APFP14: Reasoning | 241 | 13.6 | 186 | 14.6 | 55 | 10.9 | 4.45 | **.020** |

**Note.** Bold indicates significant effect of meal vs snack context on feeding practice. SPFP = Structure-based parental feeding practice; APFP = Autonomy support parental feeding practice.

**Additional file 2: Structure-based feeding practices**

**Table B.** SPFP1: Modelling - Sit and eat with your child (AIC = 964.3)

| **Model term** | **Coefficient** | **Std. Error** | ***t*** | ***p*** |
| --- | --- | --- | --- | --- |
| Intercept | -1.321 | 0.002 | -869.25 | **<.001** |
| State stress | -0.164 | 0.231 | -0.711 | .477 |
| State negative affect | 0.303 | 0.347 | 0.875 | .382 |
| State positive affect | 0.060 | 0.214 | 0.279 | .780 |
| Health-related goals |  |  |  |  |
| Goal = 0 | 0^b^ | - | - | - |
| Goal = 1 | -0.415 | 0.002 | -273.96 | **<.001** |
| Atmosphere |  |  |  |  |
| Positive / Neutral | 0.778 | 0.002 | 512.003 | **<.001** |
| Negative | 0^b^ | - | - | - |
| Parent initiated | 0.469 | 0.001 | 317.531 | **<.001** |
| Other initiated | 0^b^ | - | - | - |
| Meal | 2.098 | 0.001 | 1546.65 | **<.001** |
| Snack | 0^b^ | - | - | - |
| Probability of child avid eating | -1.243 | 0.002 | -831.22 | **<.001** |
| Parent age | 0.018 | 0.001 | 13.349 | **<.001** |
| Child age | 0.014 | 0.001 | 11.077 | **<.001** |
| Weekday | -0.216 | 0.001 | -144.95 | **<.001** |

**Note.** Paired eating occasions = 902. Bold indicates significant effect. b. This coefficient is set to zero because it is redundant.

**Table C.** SPFP2: Meal & snack routines - Choose where your child ate meal or snack (AIC = 728.7)

| **Model term** | **Coefficient** | **Std. Error** | ***t*** | ***p*** |
| --- | --- | --- | --- | --- |
| Intercept | -0.730 | 0.001 | -621.707 | **<.001** |
| State stress | 0.222 | 0.001 | 183.384 | **<.001** |
| State negative affect | -0.036 | 0.001 | -31.101 | **<.001** |
| State positive affect | 0.055 | 0.001 | 45.425 | **<.001** |
| Health-related goals |  |  |  |  |
| Goal = 0 | 0^b^ | - | - | - |
| Goal = 1 | 0.184 | 0.001 | 156.642 | **<.001** |
| Atmosphere |  |  |  |  |
| Positive / Neutral | 0.778 | 0.002 | 512.003 | **<.001** |
| Negative | 0^b^ | - | - | - |
| Parent initiated | 0.993 | 0.001 | 845.797 | **<.001** |
| Other initiated | 0^b^ | - | - | - |
| Meal | 1.718 | 0.001 | 1416.737 | **<.001** |
| Snack | 0^b^ | - | - | - |
| Probability of child avid eating | -2.158 | 0.001 | -1779.85 | **<.001** |
| Parent age | 0.051 | 0.001 | 43.473 | **<.001** |
| Child age | -0.006 | 0.001 | -5.313 | **<.001** |
| Weekday | 0.054 | 0.001 | 46.18 | **<.001** |

**Note.** Paired eating occasions = 888. Bold indicates significant effect. b. This coefficient is set to zero because it is redundant.

**Table D.** SPFP3: Food availability - Choose what foods your child got to eat (AIC = 863.3)

| **Model term** | **Coefficient** | **Std. Error** | ***t*** | ***p*** |
| --- | --- | --- | --- | --- |
| Intercept | 1.480 | 1.208 | 1.225 | .220 |
| State stress | -0.054 | 0.246 | -0.220 | .826 |
| State negative affect | -0.129 | 0.362 | -0.356 | .722 |
| State positive affect | -0.115 | 0.220 | -0.524 | .600 |
| Health-related goals |  |  |  |  |
| Goal = 0 | 0^b^ | - | - | - |
| Goal = 1 | 1.246 | 0.338 | 3.683 | **<.001** |
| Atmosphere |  |  |  |  |
| Positive / Neutral | 0.039 | 0.269 | 0.146 | .884 |
| Negative | 0^b^ | - | - | - |
| Parent initiated | 1.250 | 0.216 | 5.788 | **<.001** |
| Other initiated | 0^b^ | - | - | - |
| Meal | 0.041 | 0.233 | 0.175 | .861 |
| Snack | 0^b^ | - | - | - |
| Probability of child avid eating | -0.996 | 0.763 | -1.305 | .192 |
| Parent age | -0.024 | 0.024 | -0.997 | .319 |
| Child age | 0.002 | 0.013 | 0.115 | .909 |
| Weekday | -0.079 | 0.204 | -0.386 | .699 |

**Note.** Paired eating occasions = 899. Bold indicates significant effect. b. This coefficient is set to zero because it is redundant.

**Table E.** SPFP4: Monitoring (type) - Closely monitor the type of food eaten by your child (AIC = 916.3)

| **Model term** | **Coefficient** | **Std. Error** | ***t*** | ***p*** |
| --- | --- | --- | --- | --- |
| Intercept | -3.496 | 0.001 | -3842.582 | **<.001** |
| State stress | 0.624 | 0.001 | 686.519 | **<.001** |
| State negative affect | -0.277 | 0.262 | -1.056 | .291 |
| State positive affect | 0.269 | 0.001 | 296.117 | **<.001** |
| Health-related goals |  |  |  |  |
| Goal = 0 | 0^b^ | - | - | - |
| Goal = 1 | 1.825 | 0.001 | 2005.760 | **<.001** |
| Atmosphere |  |  |  |  |
| Positive / Neutral | -0.378 | 0.001 | -415.601 | **<.001** |
| Negative | 0^b^ | - | - | - |
| Parent initiated | 0.586 | 0.001 | 644.120 | **<.001** |
| Other initiated | 0^b^ | - | - | - |
| Meal | -0.066 | 0.001 | -72.687 | **<.001** |
| Snack | 0^b^ | - | - | - |
| Probability of child avid eating | -0.429 | 0.001 | -457.079 | **<.001** |
| Parent age | 0.110 | 0.001 | 120.020 | **<.001** |
| Child age | -0.013 | 0.001 | -14.847 | **<.001** |
| Weekday | 0.118 | 0.001 | 125.433 | **<.001** |

**Note.** Paired eating occasions = 889. Bold indicates significant effect. b. This coefficient is set to zero because it is redundant.

**Table F.** SPFP5: Monitoring (amount) - Closely monitor the amount of food eaten by your child (AIC = 958.8)

| **Model term** | **Coefficient** | **Std. Error** | ***t*** | ***p*** |
| --- | --- | --- | --- | --- |
| Intercept | -1.465 | 2.218 | -0.660 | 0.509 |
| State stress | 0.454 | 0.247 | 1.839 | **0.066** |
| State negative affect | -0.333 | 0.351 | -0.950 | 0.342 |
| State positive affect | 0.200 | 0.221 | 0.904 | 0.366 |
| Health-related goals |  |  |  |  |
| Goal = 0 | 0^b^ | - | - | - |
| Goal = 1 | 0.295 | 0.372 | 0.795 | 0.427 |
| Atmosphere |  |  |  |  |
| Positive / Neutral | -0.321 | 0.272 | -1.177 | 0.239 |
| Negative | 0^b^ | - | - | - |
| Parent initiated | 0.432 | 0.218 | 1.976 | **0.048** |
| Other initiated | 0^b^ | - | - | - |
| Meal | 0.702 | 0.253 | 2.778 | **0.005** |
| Snack | 0^b^ | - | - | - |
| Probability of child avid eating | 0.824 | 1.432 | 0.575 | 0.565 |
| Parent age | 0.013 | 0.044 | 0.292 | 0.770 |
| Child age | 0.000 | 0.024 | 0.009 | 0.992 |
| Weekday | 0.035 | 0.201 | 0.173 | 0.862 |

**Note.** Paired eating occasions = 891. Bold indicates significant effect. b. This coefficient is set to zero because it is redundant.

**Table G.** SPFP6: Guided choices - Allow your child to choose what to eat, from several options you picked out (AIC = 1172.1)

| **Model term** | **Coefficient** | **Std. Error** | ***t*** | ***p*** |
| --- | --- | --- | --- | --- |
| Intercept | -0.015 | 0.965 | -0.015 | .988 |
| State stress | 0.023 | 0.202 | 0.113 | .910 |
| State negative affect | 0.078 | 0.295 | 0.263 | .792 |
| State positive affect | 0.097 | 0.181 | 0.537 | .591 |
| Health-related goals |  |  |  |  |
| Goal = 0 | 0^b^ | - | - | - |
| Goal = 1 | -0.030 | 0.301 | -0.101 | .920 |
| Atmosphere |  |  |  |  |
| Positive / Neutral | 0.170 | 0.218 | 0.778 | .437 |
| Negative | 0^b^ | - | - | - |
| Parent initiated | -0.211 | 0.176 | -1.199 | .230 |
| Other initiated | 0^b^ | - | - | - |
| Meal | 0.184 | 0.203 | 0.905 | .366 |
| Snack | 0^b^ | - | - | - |
| Probability of child avid eating | -1.002 | 0.613 | -1.635 | .102 |
| Parent age | -0.002 | 0.019 | -0.125 | .900 |
| Child age | 0.025 | 0.011 | 2.275 | **.023** |
| Weekday | -0.158 | 0.167 | -0.949 | .343 |

**Note.** Paired eating occasions = 873. Bold indicates significant effect. b. This coefficient is set to zero because it is redundant.

**Additional file 3: Autonomy support feeding practices**

**Table H.** APFP7: Child involvement (what) - Involve your child in deciding what foods they would eat (AIC = 1011.0)

| **Model term** | **Coefficient** | **Std. Error** | ***t*** | ***p*** |
| --- | --- | --- | --- | --- |
| Intercept | 1.927 | 1.404 | 1.373 | .170 |
| State stress | -0.149 | 0.225 | -0.663 | .507 |
| State negative affect | 0.060 | 0.333 | 0.179 | .858 |
| State positive affect | -0.238 | 0.206 | -1.155 | .248 |
| Health-related goals |  |  |  |  |
| Goal = 0 | 0^b^ | - | - | - |
| Goal = 1 | -0.470 | 0.359 | -1.308 | .191 |
| Atmosphere |  |  |  |  |
| Positive / Neutral | 0.165 | 0.245 | 0.672 | .502 |
| Negative | 0^b^ | - | - | - |
| Parent initiated | -0.421 | 0.203 | -2.076 | **.038** |
| Other initiated | 0^b^ | - | - | - |
| Meal | -0.224 | 0.242 | -0.927 | .354 |
| Snack | 0^b^ | - | - | - |
| Probability of child avid eating | -0.104 | 0.902 | -0.115 | .908 |
| Parent age | -0.011 | 0.028 | -0.400 | .689 |
| Child age | 0.015 | 0.016 | 0.929 | .353 |
| Weekday | -0.631 | 0.198 | -3.184 | **.001** |

**Note.** Paired eating occasions = 888. Bold indicates significant effect. b. This coefficient is set to zero because it is redundant.

**Table I.** APFP8: Child involvement (more) - Allow your child to take more food if they asked for it (AIC = 685.3)

| **Model term** | **Coefficient** | **Std. Error** | ***t*** | ***p*** |
| --- | --- | --- | --- | --- |
| Intercept | -3.764 | 2.656 | -1.418 | .156 |
| State stress | 0.250 | 0.300 | 0.835 | .404 |
| State negative affect | 0.485 | 0.475 | 1.020 | .308 |
| State positive affect | 0.238 | 0.285 | 0.835 | .404 |
| Health-related goals |  |  |  |  |
| Goal = 0 | 0^b^ | - | - | - |
| Goal = 1 | 1.365 | 0.481 | 2.839 | **.005** |
| Atmosphere |  |  |  |  |
| Positive / Neutral | 0.648 | 0.326 | 1.986 | **.047** |
| Negative | 0^b^ | - | - | - |
| Parent initiated | 0.170 | 0.292 | 0.581 | .561 |
| Other initiated | 0^b^ | - | - | - |
| Meal | 2.698 | 0.361 | 7.470 | **<.001** |
| Snack | 0^b^ | - | - | - |
| Probability of child avid eating | -0.423 | 1.664 | -0.254 | .799 |
| Parent age | 0.033 | 0.053 | 0.620 | .535 |
| Child age | 0.023 | 0.028 | 0.829 | .407 |
| Weekday | -0.250 | 0.263 | -0.953 | .340 |

**Note.** Paired eating occasions = 777. Bold indicates significant effect. b. This coefficient is set to zero because it is redundant.

**Table J.** APFP9: Nutrition education (why more) - Teach your child about why you wanted them to eat more of certain foods (AIC = 716.7)

| **Model term** | **Coefficient** | **Std. Error** | ***t*** | ***p*** |
| --- | --- | --- | --- | --- |
| Intercept | -5.432 | 2.842 | -1.911 | .056 |
| State stress | 0.253 | 0.299 | 0.845 | .398 |
| State negative affect | 0.203 | 0.469 | 0.433 | .665 |
| State positive affect | 0.101 | 0.280 | 0.360 | .719 |
| Health-related goals |  |  |  |  |
| Goal = 0 | 0^b^ | - | - | - |
| Goal = 1 | 1.007 | 0.530 | 1.898 | .058 |
| Atmosphere |  |  |  |  |
| Positive / Neutral | 0.149 | 0.336 | 0.444 | .657 |
| Negative | 0^b^ | - | - | - |
| Parent initiated | 0.037 | 0.283 | 0.132 | .895 |
| Other initiated | 0^b^ | - | - | - |
| Meal | 0.519 | 0.340 | 1.527 | .127 |
| Snack | 0^b^ | - | - | - |
| Probability of child avid eating | 0.167 | 1.794 | 0.093 | .926 |
| Parent age | 0.052 | 0.057 | 0.901 | .368 |
| Child age | 0.013 | 0.030 | 0.414 | .679 |
| Weekday | -0.074 | 0.251 | -0.293 | .770 |

**Note.** Paired eating occasions = 818. Bold indicates significant effect. b. This coefficient is set to zero because it is redundant.

**Table K.** APFP10: Nutrition education (why less) - Teach your child about why you wanted them to eat less of certain foods (AIC = 633.4)

| **Model term** | **Coefficient** | **Std. Error** | ***t*** | ***p*** |
| --- | --- | --- | --- | --- |
| Intercept | -6.341 | 3.105 | -2.042 | **.041** |
| State stress | 0.002 | 0.329 | 0.007 | .994 |
| State negative affect | -0.079 | 0.512 | -0.154 | .878 |
| State positive affect | 0.065 | 0.312 | 0.207 | .836 |
| Health-related goals |  |  |  |  |
| Goal = 0 | 0^b^ | - | - | - |
| Goal = 1 | -0.945 | 0.532 | -1.778 | .076 |
| Atmosphere |  |  |  |  |
| Positive / Neutral | -0.081 | 0.369 | -0.221 | .825 |
| Negative | 0^b^ | - | - | - |
| Parent initiated | 0.165 | 0.305 | 0.541 | .589 |
| Other initiated | 0^b^ | - | - | - |
| Meal | 0.229 | 0.357 | 0.643 | .520 |
| Snack | 0^b^ | - | - | - |
| Probability of child avid eating | 0.142 | 1.915 | 0.074 | .941 |
| Parent age | 0.090 | 0.062 | 1.433 | .152 |
| Child age | 0.025 | 0.033 | 0.773 | .439 |
| Weekday | 0.060 | 0.273 | 0.221 | .825 |

**Note.** Paired eating occasions = 822. Bold indicates significant effect. b. This coefficient is set to zero because it is redundant.

**Table L.** APFP11: Encouragement - Encourage your child to try at least a small amount of all foods offered (AIC = 720.8)

| **Model term** | **Coefficient** | **Std. Error** | ***t*** | ***p*** |
| --- | --- | --- | --- | --- |
| Intercept | -1.821 | 0.002 | -1140.918 | **<.001** |
| State stress | 0.195 | 0.002 | 122.474 | **<.001** |
| State negative affect | -0.779 | 0.002 | -472.603 | **<.001** |
| State positive affect | -0.149 | 0.002 | -93.466 | **<.001** |
| Health-related goals |  |  |  |  |
| Goal = 0 | 0^b^ | - | - | - |
| Goal = 1 | 1.421 | 0.002 | 861.840 | **<.001** |
| Atmosphere |  |  |  |  |
| Positive / Neutral | -0.100 | 0.002 | -62.446 | **<.001** |
| Negative | 0^b^ | - | - | - |
| Parent initiated | 0.524 | 0.002 | 327.899 | **<.001** |
| Other initiated | 0^b^ | - | - | - |
| Meal | 2.035 | 0.002 | 1274.594 | **<.001** |
| Snack | 0^b^ | - | - | - |
| Probability of child avid eating | -1.136 | 0.002 | -711.510 | **<.001** |
| Parent age | -0.031 | 0.002 | -20.064 | **<.001** |
| Child age | 0.008 | 0.001 | 5.149 | **<.001** |
| Weekday | 0.026 | 0.002 | 16.040 | **<.001** |

**Note.** Paired eating occasions = 701. Bold indicates significant effect. b. This coefficient is set to zero because it is redundant.

**Table M.** APFP12: Negotiate (amount) - Negotiate with your child about how much food to eat (AIC = 876.2)

| **Model term** | **Coefficient** | **Std. Error** | ***t*** | ***p*** |
| --- | --- | --- | --- | --- |
| Intercept | -2.411 | 1.618 | -1.490 | .136 |
| State stress | 0.444 | 0.242 | 1.834 | .067 |
| State negative affect | -0.436 | 0.367 | -1.189 | .234 |
| State positive affect | 0.153 | 0.228 | 0.669 | .503 |
| Health-related goals |  |  |  |  |
| Goal = 0 | 0^b^ | - | - | - |
| Goal = 1 | -0.019 | 0.401 | -0.046 | .963 |
| Atmosphere |  |  |  |  |
| Positive / Neutral | -0.532 | 0.257 | -2.069 | **.039** |
| Negative | 0^b^ | - | - | - |
| Parent initiated | 0.129 | 0.224 | 0.576 | .564 |
| Other initiated | 0^b^ | - | - | - |
| Meal | 1.093 | 0.284 | 3.851 | **<.001** |
| Snack | 0^b^ | - | - | - |
| Probability of child avid eating | 1.032 | 1.046 | 0.987 | .324 |
| Parent age | -0.002 | 0.033 | -0.076 | .939 |
| Child age | 0.002 | 0.018 | 0.118 | .906 |
| Weekday | 0.110 | 0.208 | 0.527 | .598 |

**Note.** Paired eating occasions = 820. Bold indicates significant effect. b. This coefficient is set to zero because it is redundant.

**Table N.** APFP13: Negotiate (what) - Negotiate with your child about what food they eat (AIC = 875.7)

| **Model term** | **Coefficient** | **Std. Error** | ***t*** | ***p*** |
| --- | --- | --- | --- | --- |
| Intercept | -1.699 | 1.936 | -0.878 | .380 |
| State stress | 0.307 | 0.248 | 1.237 | .216 |
| State negative affect | -0.670 | 0.368 | -1.820 | .069 |
| State positive affect | 0.052 | 0.232 | 0.224 | .823 |
| Health-related goals |  |  |  |  |
| Goal = 0 | 0^b^ | - | - | - |
| Goal = 1 | 0.538 | 0.389 | 1.381 | .167 |
| Atmosphere |  |  |  |  |
| Positive / Neutral | -0.701 | 0.263 | -2.664 | **.008** |
| Negative | 0^b^ | - | - | - |
| Parent initiated | 0.100 | 0.232 | 0.432 | .666 |
| Other initiated | 0^b^ | - | - | - |
| Meal | -0.009 | 0.271 | -0.034 | .973 |
| Snack | 0^b^ | - | - | - |
| Probability of child avid eating | 0.231 | 1.247 | 0.185 | .853 |
| Parent age | 0.010 | 0.039 | 0.259 | .795 |
| Child age | 0.005 | 0.021 | 0.217 | .828 |
| Weekday | -0.142 | 0.205 | -0.693 | .488 |

**Note.** Paired eating occasions = 835. Bold indicates significant effect. b. This coefficient is set to zero because it is redundant.

**Table O.** APFP14: Reasoning - Tell your child you wanted them to eat less of certain foods (AIC = 505.4)

| **Model term** | **Coefficient** | **Std. Error** | ***t*** | ***p*** |
| --- | --- | --- | --- | --- |
| Intercept | -6.838 | 3.905 | -1.751 | .080 |
| State stress | 0.189 | 0.355 | 0.532 | .595 |
| State negative affect | -0.144 | 0.577 | -0.249 | .804 |
| State positive affect | 0.081 | 0.364 | 0.224 | .823 |
| Health-related goals |  |  |  |  |
| Goal = 0 | 0^b^ | - | - | - |
| Goal = 1 | -0.748 | 0.599 | -1.250 | .211 |
| Atmosphere |  |  |  |  |
| Positive / Neutral | -0.478 | 0.403 | -1.186 | .236 |
| Negative | 0^b^ | - | - | - |
| Parent initiated | -0.421 | 0.354 | -1.189 | .234 |
| Other initiated | 0^b^ | - | - | - |
| Meal | 0.264 | 0.416 | 0.635 | .525 |
| Snack | 0^b^ | - | - | - |
| Probability of child avid eating | 1.685 | 2.643 | 0.637 | .524 |
| Parent age | 0.074 | 0.078 | 0.943 | .346 |
| Child age | 0.012 | 0.042 | 0.288 | .773 |
| Weekday | -0.472 | 0.314 | -1.502 | .133 |

**Note.** Paired eating occasions = 852. Bold indicates significant effect. b. This coefficient is set to zero because it is redundant.
